# The Peninsular Journal

**Published:** 1857-02

**Authors:** 


*The Peninsular Journal.*—From time to time—we believe in every number—the "Peninsular" favors us with a notice, usually in the form of a complaint, at our "repeated and unprovoked attacks." Having a defective memory, we have felt somewhat conscience smitten whenever reminded of this our fault. With the view of making any reparation in our power, we have just taken the pains to examine our files, and ascertain wherein we have sinned. On careful inspection, we find that we have not mentioned the "Peninsular," in any manner, since May last; and then only to express our gratification at the failure of the suit against the University of Michigan.

And now will the "Peninsular" be so kind as to discontinue this schoolboy whimpering, and inform us what charges it has against us. We shall then be in a condition to answer. A faint hint lugged into its notice of Prof. Hamilton's Fracture Report, leads us to surmise that its last grievance is a correspondence between two parties, neither of them directly connected with either of the journals. If we are correct in this, we will, with the consent of the party most interested, obtain and print this correspondence, and allow the profession to judge between us. So far as we understand it, one party asked the other to discontinue an illegal and dishonorable course of conduct —the penalty for which is five years in the States Prison. This reasonable request met with a refusal. Does the "Peninsular" wish the correspondence published?

In its last number, the "Peninsular" appeals to the profession at large to share in its quarrel with the Medical Independent. Under the present circumstances we respectfully decline, so far as we may have been included in that appeal. The parties to the libel suit in question, are both medical gentlemen and both editors; but here their equality ceases. One is at home, wealthy, surrounded by friends, powerful. The other is a foreigner, a man of the closet, a student whose name is only known in connection with his reputation in natural science. He is poor, a stranger, powerless. Let the law decide impartially between these men. We will not risk an opinion.

